# Influence of low back pain and its remission on motor abundance in a low-load lifting task

**DOI:** 10.1038/s41598-020-74707-4

**Published:** 2020-10-20

**Authors:** Bernard X. W. Liew, Alessandro Marco De Nunzio, Shraddha Srivastava, Deborah Falla

**Affiliations:** 1 School of Sport, Rehabilitation and Exercise Sciences, University of Essex, Colchester, CO4 3SQ Essex UK; 2 LUNEX International University of Health, Exercise and Sports, 50, avenue du Parc des Sports, 4671 Differdange, Luxembourg; 3 Department of Health Sciences and Research, College of Health Professions, Medical University of South Carolina, 77 President Street, Charleston, SC 29425 USA; 4 Centre of Precision Rehabilitation for Spinal Pain (CPR Spine), School of Sport, Exercise and Rehabilitation Sciences, University of Birmingham, Edgbaston, B152TT UK

**Keywords:** Neuroscience, Biomarkers, Health care, Medical research

## Abstract

Having an abundance of motor solutions during movement may be advantageous for the health of musculoskeletal tissues, given greater load distribution between tissues. The aim of the present study was to understand whether motor abundance differs between people with and without low back pain (LBP) during a low-load lifting task. Motion capture with electromyography (EMG) assessment of 15 muscles was performed on 48 participants [healthy control (con) = 16, remission LBP (rLBP) = 16, current LBP (cLBP) = 16], during lifting. Non-negative matrix factorization and uncontrolled manifold analysis were performed to decompose inter-repetition variability in the temporal activity of muscle modes into goal equivalent (GEV) and non-goal equivalent (NGEV) variabilities in the control of the pelvis and trunk linear displacements. Motor abundance occurs when the ratio of GEV to NGEV exceeds zero. There were significant group differences in the temporal activity of muscle modes, such that both cLBP and rLBP individuals demonstrated greater activity of muscle modes that reflected lumbopelvic coactivation during the lifting phase compared to controls. For motor abundance, there were no significant differences between groups. Individuals with LBP, including those in remission, had similar overall motor abundance, but use different activation profiles of muscle modes than asymptomatic people during lifting.

## Introduction

Low back pain (LBP) is a highly prevalent disorder and it ranks as the number one cause of years lived with disability^1^. Approximately 28% of individuals with a recent onset of LBP experience incomplete recovery at 12 months^2^. For individuals who experience symptom resolution from LBP, altered trunk muscle activation may persist^3^; and this has been thought to increase the risk of symptom recurrence^3,4^. Lifting is an ideal task to investigate in individuals with LBP, as it commonly provokes pain^5^ and functional alterations in people with LBP during lifting may persist into remission^6,7^.

Individuals with LBP have been reported to lift with 11% greater compression and 18% greater shear spinal loads than asymptomatic controls^8^. Simple kinematic measures such as linear distance of the load from the L5/S1 disc centroid, and trunk flexion angle, are predictive of spinal loads during lifting^9^. The further the horizontal distance of the load from the spine, and the greater the trunk flexion angle, the greater the load on the spine^9^. It is possible that individuals with LBP seek to minimize variation of the pelvis and trunk positions during repeated lifts, since such variability may increase the proportion of lifts that result in high spinal loads^10^.

There is an abundance in the number of muscles available to perform any movement^11^. Rather than controlling the activation of individual muscles during movement, it has been thought that the nervous system only controls deviations of muscular behaviour that perturb the movement goal—termed non-goal equivalent (NGE) deviations^12^. Deviations of muscular behaviour that do not affect the movement goal, due to their cancelling effect, remain free to vary—termed goal equivalent (GE) deviations^12^. To this end, the Uncontrolled Manifold Analysis (UCM) provides a powerful method to quantify the proportion of the variability in muscular behaviour between repeated performances that result in GE deviations (GEV) and those that result in NGE deviations (NGEV).

A greater logarithmic ratio of GEV to NGEV, termed motor abundance^13^, may be advantageous for spinal health given that more movement solutions are available to achieve the same goal. Greater GEV may increase the load distribution between muscles during lifting, potentially reducing tissue loading, and minimizing muscular fatigue^14^. Feedback and practice training is commonly prescribed to manage LBP, and such training is designed to reduce NGEV^15^. Hence, knowledge of how LBP influences the relative proportioning of GEV and NGEV during movement would help inform the design of such training programs.

Although there are many studies which have investigated how LBP alters the activity of single muscles^7,16–18^, no studies have evaluated how LBP alters motor abundance towards the stabilization (defined as reducing the variability) of important kinematic profiles during lifting. Only two studies have used UCM to investigate motor control impairments in people with LBP using segmental angles as motor degrees of freedom (DOF), to stabilize the centre of mass (COM) and head trajectory displacements during a surface perturbation in standing^19^ and during a sit-to-stand task^20^. One study reported that individuals with LBP had similar motor abundance, GEV, and NGEV as controls^19^. The other study found that individuals with LBP had a smaller GEV and a greater NGEV compared to healthy controls^20^. It may be that greater motor control impairments in LBP manifest in tasks with greater spinal flexion demands, such as that involved during sit-to-stand compared to static standing.

The aim of the present study is to understand differences in motor abundance, and its constituent components of GEV and NGEV, towards the control of the pelvis and trunk positions during lifting, in individuals with current LBP (cLBP), those in remission (rLBP), and healthy controls (con). Given previous results^20^, we hypothesized that individuals with cLBP will have smaller motor abundance than individuals with rLBP and controls, and that rLBP individuals will have smaller abundance than controls. We also hypothesized that a lower motor abundance would be due to smaller GEV, and a greater NGEV. Lastly, we hypothesized that individuals with cLBP would have a greater magnitude of muscle groups (termed as “modes”, see Materials and Methods for definition) that reflect greater trunk extensor muscle activity^6,21^, compared to individuals with rLBP and controls.

## Materials and methods

### Study design

This was a cross-sectional study conducted at the Centre of Precision Rehabilitation for Spinal Pain, University of Birmingham, United Kingdom. All participants provided written informed consent. Reporting of the present study adheres to the STROBE guidelines^22^.

### Selection criteria

Participants aged between 18 to 55 years with adequate conversational English language, were invited to volunteer. Participants were eligible to be included into one of three groups, based on the following criteria:

- cLBP: present episode of LBP lasting > 24 h, with a minimum intensity on the numerical rating scale (NRS) score ≥ 2/10 (where 0 = no pain, 10 = being maximal pain)^23^.
- rLBP: presently in symptom remission from a LBP episode experienced within the last year, with an NRS score ≤ 1/10.
- con: No relevant history of LBP that limited their function and/or required treatment from a health professional in the past year.

Participants were excluded if they had previous spinal fracture, spinal surgery, rheumatologic, metabolic, or infectious conditions as self-reported, inability to perform at least 10 full spinal flexion repetitions on screening, and pregnancy.

### Experimental task

Participants performed repeated low load (7% body weight [BW])^24^ lifting of a basket (30 × 36 × 10 cm) from the ground, with the mid-point of the basket’s handle positioned 25 cm forward horizontally from the mid-point of the foot on the ground^25^ (see supplementary for schematic figure task). Lifting was performed barefooted with a 30 cm intra-malleoli distance, and with a freely selected toe-out angle^26^. Participants were instructed verbatim—“lift in a way that is most comfortable”, to bimanually lift the basket in a symmetrical way^27^. Participants’ entire foot had to keep contact with the ground, and they had to maintain a consistent lifting style throughout the task. Participants performed six lifting sets of five consecutive repetitions, after a familiarisation of 10 repetitions, with an inter-set rest period of 5 min. Lifting was performed at a self-determined pace, which was determined during familiarization, and subsequently fixed using a metronome.

### Assessment

Twelve retroreflective 14 mm markers were placed over the bilateral anterior and posterior superior iliac spines, acromion, 1st and 5th metatarsophalangeal (MTP) joints, and posterior calcaneus. Marker trajectories were captured with eight motion capture cameras sampling at 250 Hz (BTS SMART-DX 6000, BTS Bioengineering Corp, Italy). In accordance with the SENIAM guidelines, the skin was shaved, gently abraded, and wiped with alcohol before EMG electrodes placement^28^. Fifteen wireless EMG electrode pairs (1000 Hz; BTS FreeEMG, BTS Bioengineering Corp, Italy) were placed unilaterally on the biceps brachii (BicepsB), anterior deltoid (AntDelt), latissimus dorsi (LatsD) (lateral to T9 over the muscle belly)^29^, external oblique (EO) (15 cm lateral to the umbilicus)^29^, rectus abdominis (RA) (3 cm lateral to umbilicus)^30^, iliocostalis lumborum (Ileoc) (1 cm lateral to the L5 spinous process)^31^, longissimus thoracis pars thoracis (Longis) (5 cm lateral to the T9 spinous process)^31^, soleus (Sol), lateral gastrocnemius (GL), tibialis anterior (TA), vastus lateralis (VL), rectus femoris (RF), semitendinosus (ST), biceps femoris (BicepsF), gluteus maximus (GMax). The side for electrode placements was on the right for controls, and on the side of previous/current pain for the LBP groups.

### Data processing

A virtual “pelvis” landmark was calculated using the proximal endpoint of the modelled inertial pelvic segment^32,33^, respectively. A virtual global coordinate system was created with three virtual landmarks: the origin at the mid-point of bilateral calcanei marker projected onto the floor, the mid-point of the bilateral 1st MTP marker projected onto the floor, and a landmark projected 10 cm vertically above the origin. The vertical and anterior–posterior linear displacements of the right acromion (RACR) target and pelvis landmark were calculated relative to the virtual coordinate system. Marker trajectories were filtered with a low pass, zero-lag, 4th Order, Butterworth (6 Hz). The RACR target and pelvis landmark were used as a measure of trunk and pelvis displacements, respectively.

To remove the electrocardiogram artefact, EMG signals were high pass filtered^34^, by transforming the signals to the frequency domain through the Fast Fourier Transform (FFT), removing the spectral components below 40 Hz, transforming back to time–space through the Inverse FFT. Subsequently, the signals were rectified and low pass filtered with a zero-lag, 4th Order, Butterworth (5 Hz)^35^. The maximal EMG amplitude of each muscle per repetition was extracted and averaged within a set, to be used as normalizing factor^36^. The RA EMG signals were excluded due to movement artefacts repeatedly occuring during trunk flexion as perfect and constant adherence of the electrodes to the skin could not always be granted.

A lifting repetition was defined from the instant the load left the ground, to a fully upright position, and back to the ground. A lifting repetition was divided into two phases: (1) a lifting phase starting when the positive-vertical velocity of the right acromion marker exceeded 10%, and ended when it dropped below 10%, both of the peak vertical velocity during each set; (2) a lowering phase starting when the negative-vertical velocity of the right acromion marker exceeded 10%, and ended when it dropped below 10%, both of its peak vertical velocity during each set. Segmentation of the kinematic and EMG signals were undertaken independently for each lifting and lowering phase, and were time-normalized to 100 datapoints. Twenty cycles from all participants, for each of the lifting and lowering phases of all EMG channels and kinematic data, were free from artefacts, and these were used for the UCM analysis. One individual with cLBP was removed, as 20 cycles of data were not available, due to significant movement artefacts from multiple EMG electrodes arising from poor electrode–skin adherence.

### UCM analysis—part 1 (muscle mode extraction)

NMF to identify muscle modes was performed^37^, per participant, across both lifting and lowering phases. We note that although muscle mode extraction is commonly performed in the context of quantifying muscle synergies using UCM, in itself is not explicitly required – especially when investigating a smaller subset of regionally distinct muscles.

The processed EMG data from all repetitions were concatenated to create a 14 muscle × 2000 cycle points (100 datapoints × 20 cycles) matrix as data input. NMF factorizes the concatenated original EMG data into two matrices. W is the matrix specifiying the relative contribution of each muscle to a muscle mode, with the total contribution of all muscles to a mode summing to one^37^. H is the matrix specifying the temporal activity of each muscle mode. NMF analysis was iterated by varying the number of modes between 1 and 14. Three modes were retained as it was the lowest number that accounted for more than 90% of the VAF, while adding an additional mode did not increase VAF by more than 3%^38^ (see supplementary for illustration of VAF against mode number). Using Pearson’s correlation coefficients, modes from each participant which had the highest correlation value with that of a randomly selected reference healthy participant were matched together^39^. Since the W weights of all 14 muscles per mode sum to 1, a threshold was determined that a muscle was contributing significantly to a mode if its W weight was greater than an average of 0.071 (1/14).

### UCM analysis—part 2 (Jacobian)

At each 1% cycle, linear relations across the 20 repetitions between changes in the mean-free magnitudes of both the H weights (${\Delta {\varvec{H}}}_{20x3}$) and displacements (20 × 1 matrix) were assumed. These relationships were modelled using multiple regression of the form below:

for the pelvis displacements:

$${\Delta Pelvis }_{AP}= {\beta }_{1AP}{\Delta {\varvec{H}}}_{1}+ {\beta }_{2AP}{\Delta {\varvec{H}}}_{2} + {\beta }_{3AP}{\Delta {\varvec{H}}}_{3}$$

$${\Delta Pelvis }_{V}= {\beta }_{1V}{\Delta {\varvec{H}}}_{1}+ {\beta }_{2V}{\Delta {\varvec{H}}}_{2} + {\beta }_{3V}{\Delta {\varvec{H}}}_{3}$$

and for the trunk displacements:

$${\Delta Trunk }_{AP}= {\beta }_{1AP}{\Delta {\varvec{H}}}_{1}+ {\beta }_{2AP}{\Delta {\varvec{H}}}_{2} + {\beta }_{3AP}{\Delta {\varvec{H}}}_{3}$$

$${\Delta Trunk }_{V}= {\beta }_{1V}{\Delta {\varvec{H}}}_{1}+ {\beta }_{2V}{\Delta {\varvec{H}}}_{2} + {\beta }_{3V}{\Delta {\varvec{H}}}_{3}$$

The coefficients from the regressions were arranged into two Jacobian (${{\varvec{J}}}_{2x3}$) matrices, one for each of the outcomes of pelvis and trunk displacements:

$${\varvec{J}} = \begin{array}{ccc}{\beta }_{1AP}& {\beta }_{2AP}& {\beta }_{3AP}\\ {\beta }_{1V}& {\beta }_{2V}& {\beta }_{2V}\end{array}$$

Linear regressions to elicit ${\varvec{J}}$ were performed for each participant, each phase, and each 1% cycle.

### UCM analysis—part 3

In the current study, changes in the H weights are the DOFs (n = 3), while changes in pelvis and trunk displacements represent the outcomes (d = 2, each). UCM analysis was performed separately on each participant, each lifting phase, each 1% cycle, and for each of the two outcomes. The variance of the mean free H weights (${\Delta {\varvec{H}}}_{20x3}$) at each 1% cycle that did not affect the pelvis and trunk displacements (GEV), normalized by the DOFs in the GEV subspace (d = 1), was computed:

$$GEV= \frac{trace \left({null \left({\varvec{J}}\right)}^{T}\bullet {\varvec{C}}\bullet null \left({\varvec{J}}\right)\right)}{n-d}$$

and the variance of $\Delta {\varvec{H}}$ that perturbed the displacements (NGEV), normalized by the DOFs in the NGEV subspace (d = 2), was calculated as:

$$NGEV= \frac{trace \left({orth({{\varvec{J}}}^{{\varvec{T}}})}^{T}\bullet {\varvec{C}}\bullet orth \left({{\varvec{J}}}^{{\varvec{T}}}\right)\right)}{d}$$

with ${{\varvec{C}}}_{3{\varvec{x}}3}$ being the covariance matrix of $\Delta {\varvec{H}}.$ An index of motor abundance (IMA) was calculated as follows^13^:

$$IMA=\mathrm{log}\left(\frac{GEV}{NGEV}\right)$$

An IMA > 0 indicates that the between mode variability leaves the movement goal invariant. An IMA < 0 indicates that the between-mode variability overall perturbs the movement goal. Each of the two phases (lifting, lowering) was split into an early and late epoch, as indicated by the first and second 50% cycle points, respectively. IMA values over the early and late epochs of both the lifting and lowering phases were averaged, for subsequent statistical analysis.

### Statistical inference

All NMF, UCM, and statistical analyses were performed in R software (version 3.6.0), and codes and data can be found in a public repository^40^.

$${H}_{ijk} = \alpha + {group}_{i} + {phase}_{j }+ {group:phase}_{ij }+{ {f}_{ij}({cycle}_{k}) + subj}_{i} + {\epsilon }_{ijk}$$

The H weight of subject *i* undergoing phase *j*, at *k*% cycle can be modelled as an intercept $\alpha$, a group effect ${group}_{i}$, a task effect ${task}_{j}$, a group-by-phase interaction effect ${group:phase}_{ij}$, a smooth cycle effect ${f}_{ij}({cycle}_{k})$(defined as 15 cubic regression splines) with different shape for each level of the group-by-phase interaction effect; a subject-specific random intercept ${subj}_{i}$ and ${\epsilon }_{ijk}$ is the unexplained information, using Generalized Additive Modelling (GAM)^41 ^ (see supplementary for reasoning and potential limitation of using GAM).

The smoothing parameter that determines the “wiggliness” of the non-linear effect of time was estimated using a generalized cross-validation approach^42^. The contrast cycle difference (CCD) approach was used for statistical inference^43^, whereby the predicted pairwise group mean difference of H weights over time ($\widehat{\delta }$) for each phase, and its 95%CI, was calculated:

$$\widehat{\delta } \pm {C}_{\alpha /2}\sqrt{\widehat{var(\delta )}}$$

where ${C}_{\alpha /2}$ is a critical value for a level of $\alpha$ significance test, and $\widehat{var(\delta )}$ is the variance of the predicted $\widehat{\delta }$. A Bonferroni correction of the 95%CI confidence was performed to account for the multiple comparisons.

$$\widehat{\delta } \pm {C}_{\alpha /2g}\sqrt{\widehat{var(\delta )}}$$

where g is the number of comparisons (g = 6). Significance was defined when the adjusted 95% CI of the mean pairwise group CCD does not contain zero^43^.

Both GEV and NGEV were log-transformed prior to statistical inference^13^. For each outcome (pelvic and trunk displacements), the primary analyses involved linear mixed-effects modelling with fixed effects of group, phase, epoch, their interactions, with a subject-specific intercept, to predict the dependent variables of IMA, GEV, and NGEV. The p-values for the main and interaction effects of the mixed models were adjusted using the Benjamini-Hochberg-Yekuteli method (n = 6). Appropriate secondary post-hoc pairwise contrasts using Estimated Marginal Means, were performed when the primary main or interaction effects were significant. Significant differences were determined with a P < 0.05.

### Ethical approval

Ethical approval was obtained from the Human Research Ethics Committee of the University of Birmingham, UK (ERN_17-1717).

## Results

Demographic and clinical characteristics of the 48 participants (16 control, 16 rLBP, 16 cLBP), are reported in Table 1. The group average kinematic, original and reconstructed (from extracted modes) EMG profiles can be found in the supplementary material.

**Table 1:** Mean (standard deviation) of demographic and pain-related characteristics.

	Control (n = 16)	rLBP (n = 16)	cLBP (n = 16)
Female/male	9/7	6/10	12/4^+^
Age [years]	29.94 (9.61)	38.69 (10.54) *	40.63 (10.76)*
Weight [kg]	68.48 (13.65)	71.18 (11.49)	71.81 (12.85)
Height [cm]	168.38 (6.61)	171.69 (11.22)	168.72 (8.03)
Pain [NRS: 0 no pain–10 max pain]	–	1 (0)	4.06 (1.94) ^+^
ODI [0 no disability to 50 max disability]	–	2.69 (3.11)	10.75 (6.34) ^+^
TSK [17 no fear–68 max fear]	–	36.25 (5.66)	39.38 (5.98)
PASS-20 [0 no anxiety to 200 max anxiety]	–	31.19 (19.79)	35.38 (18.53)
Lifting phase duration [s]	1.05 (0.13)	1.11 (0.12)	1.14 (0.20)
Lowering phase duration [s]	1.13 (0.16)	1.18 (0.14)	1.23 (0.28)

*NRS* Numerical rating scale, *TSK* Tampa Scale for Kinesiophobia, *PASS-20* Pain Anxiety Symptoms Scale-20 (PASS-20), *ODI* Oswestry Disability Index.

*Significantly different from Control.

^+^Significantly different from rLBP.

### Muscle modes

**Figure 1:**
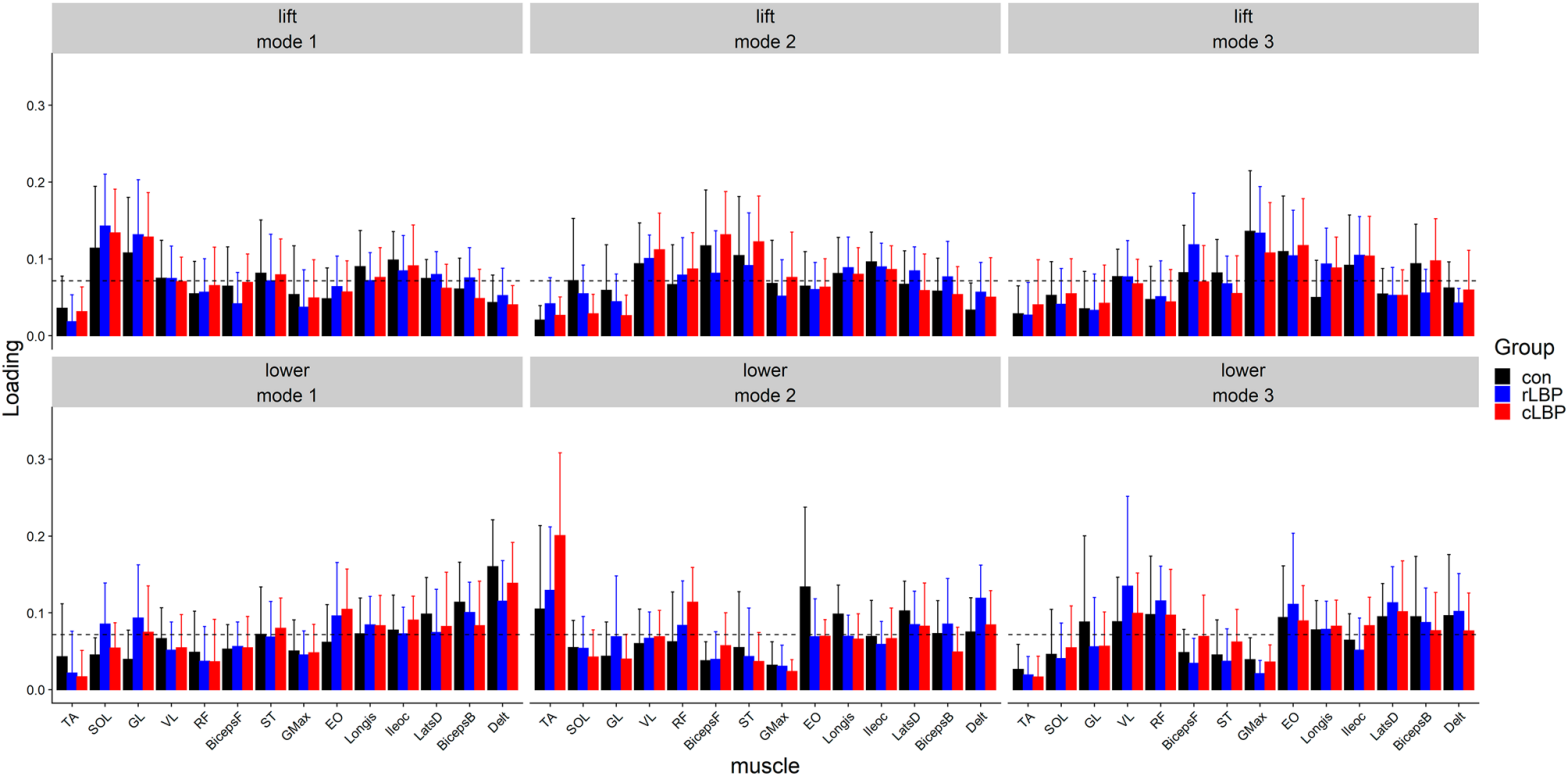
Group mean (error bar represent one standard deviation) W weights for each muscle on each muscle mode, and lifting and lowering phases. Dashed (–-) lines reflect the group mean W weights across all muscles i.e. the threshold value 0.071. *TA* tibialis anterior, *SOL* soleus, *GL* gastrocnemius lateralis, *VL* vastus lateralis, *RF* rectus femoris, *BicepsF* biceps femoris, *ST* semitendinosus, *GMax* gluteus maximus, *EO* external oblique, *Longis* longissimus thoracis pars thoracis, *Illeoc* iliocostalis lumborum, *LatsD* latissimus dorsi, *BicepsB* biceps brachii, *Delt* deltoids, *con* control, rLBP = remission low back pain, cLBP = current low back pain.

Based on the W weights for each mode in the lifting phase, mode 1 reflects a dominant ankle extensor pattern, mode 2 reflects a dominant hip–knee extensor pattern, and mode 3 reflects a dominant lumbopelvic coactivation pattern. In the lowering phase, mode 1 reflects a dominant upper limb flexor pattern, mode 2 reflects a dominant ankle flexor pattern, and mode 3 reflects a mixed multi-segmental pattern (Fig. 1).

### H weights

**Figure 2:**
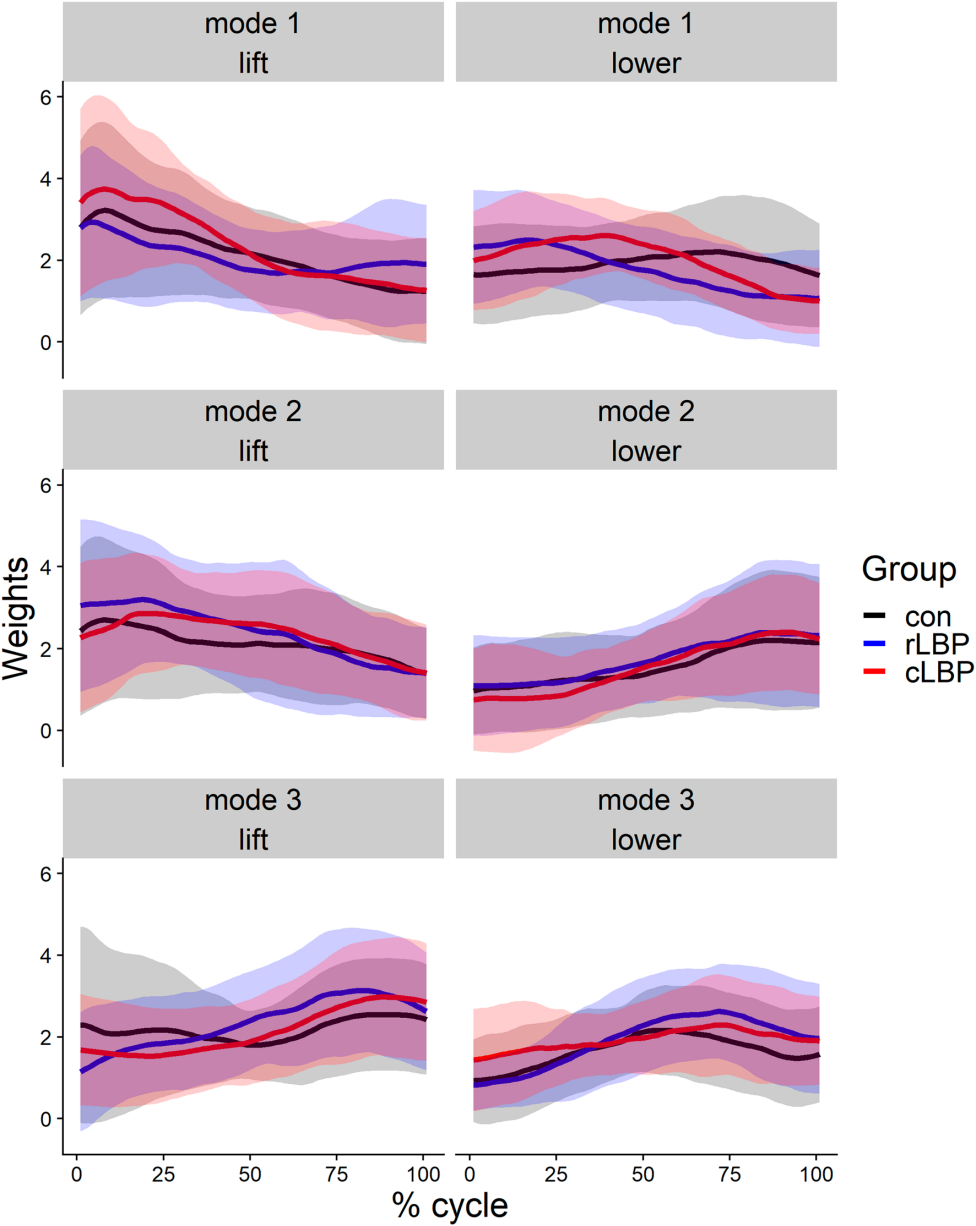
Group mean (error clouds represent one standard deviation) H weights (temporal activity) for each muscle mode and, lifting and lowering phases. *Con* control *rLBP* remission low back pain, *cLBP* current low back pain.

**Figure 3:**
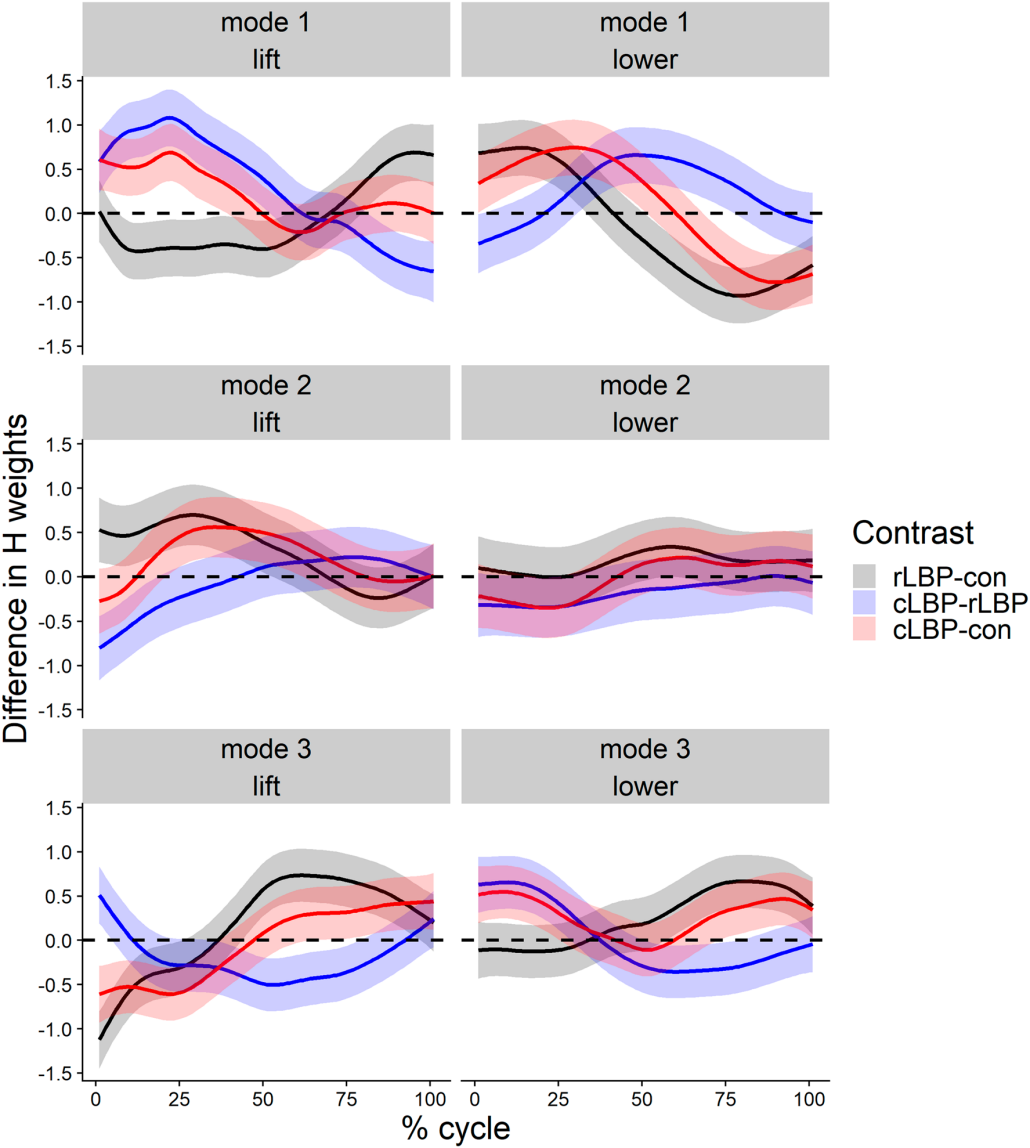
Between group mean difference (error clouds represent Bonferroni adjusted 95% confidence interval from a t distribution with 47 degrees of freedom) in H weights (temporal activity) for each muscle mode, and lifting and lowering phases. *Con* control, *rLBP* remission low back pain, *cLBP* current low back pain.

For simplicity, only the maximal of the pairwise absolute difference between groups are reported in the manuscript, with the full time-varying effect reported in Fig. 3. In the lifting phase, the biggest between differences in H magnitude were between cLBP vs rLBP (22% cycle, 1.08 [95% CI 0.75–1.40]) for mode 1, between cLBP vs rLBP (1% cycle, − 0.80 [95% CI − 1.16 to − 0.44]) for mode 2, and between rLBP vs con (1% cycle, − 1.12 [95% CI − 1.45 to − 0.80]) for mode 3 (Figs. 2, 3). In the lowering phase, the biggest between difference in H weights were between rLBP vs con (79% cycle, − 0.93 [95% CI − 1.24 to − 0.61]) for mode 1, between cLBP vs con (22% cycle, − 0.35 [95% CI − 0.69 to − 0.008]) for mode 2, and between rLBP vs con (81% cycle, 0.67 [95% CI 0.37 to 0.97]) for mode 3 (Figs. 2, 3).

### IMA, GEV, NGEV

**Figure 4:**
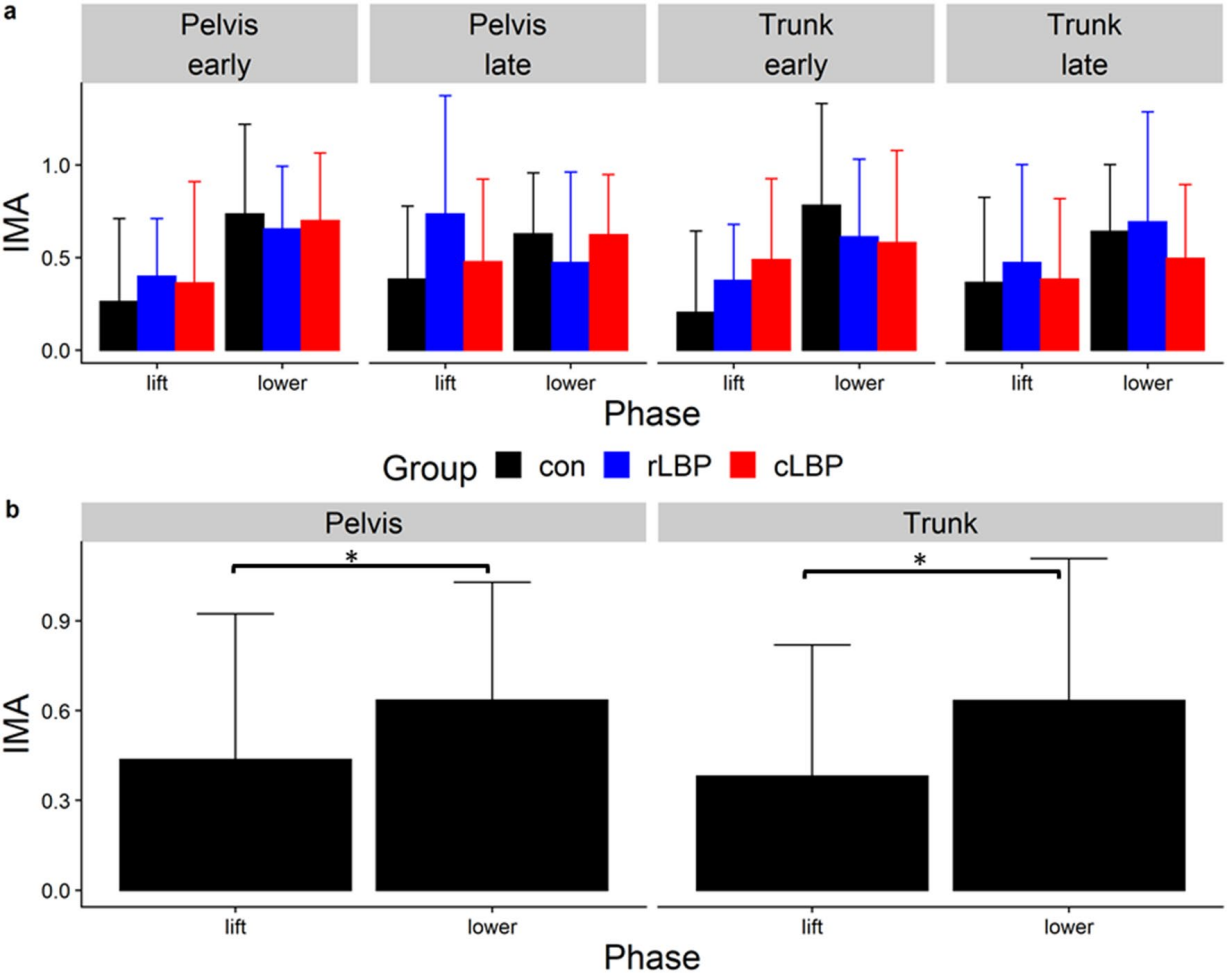
(**a**) Group mean (error bars represent one standard deviation [SD]) Index of Motor Abundance (IMA) of each segment displacement, split by phase (lift, lower) and epoch (early, late); (**b**) mean (SD) IMA averaged across all participants split by segment displacement (pelvis, trunk) and phase (lift, lower). *Con* control, *rLBP* remission low back pain, *cLBP* current low back pain. * indicates P < 0.05.

**Figure 5:**
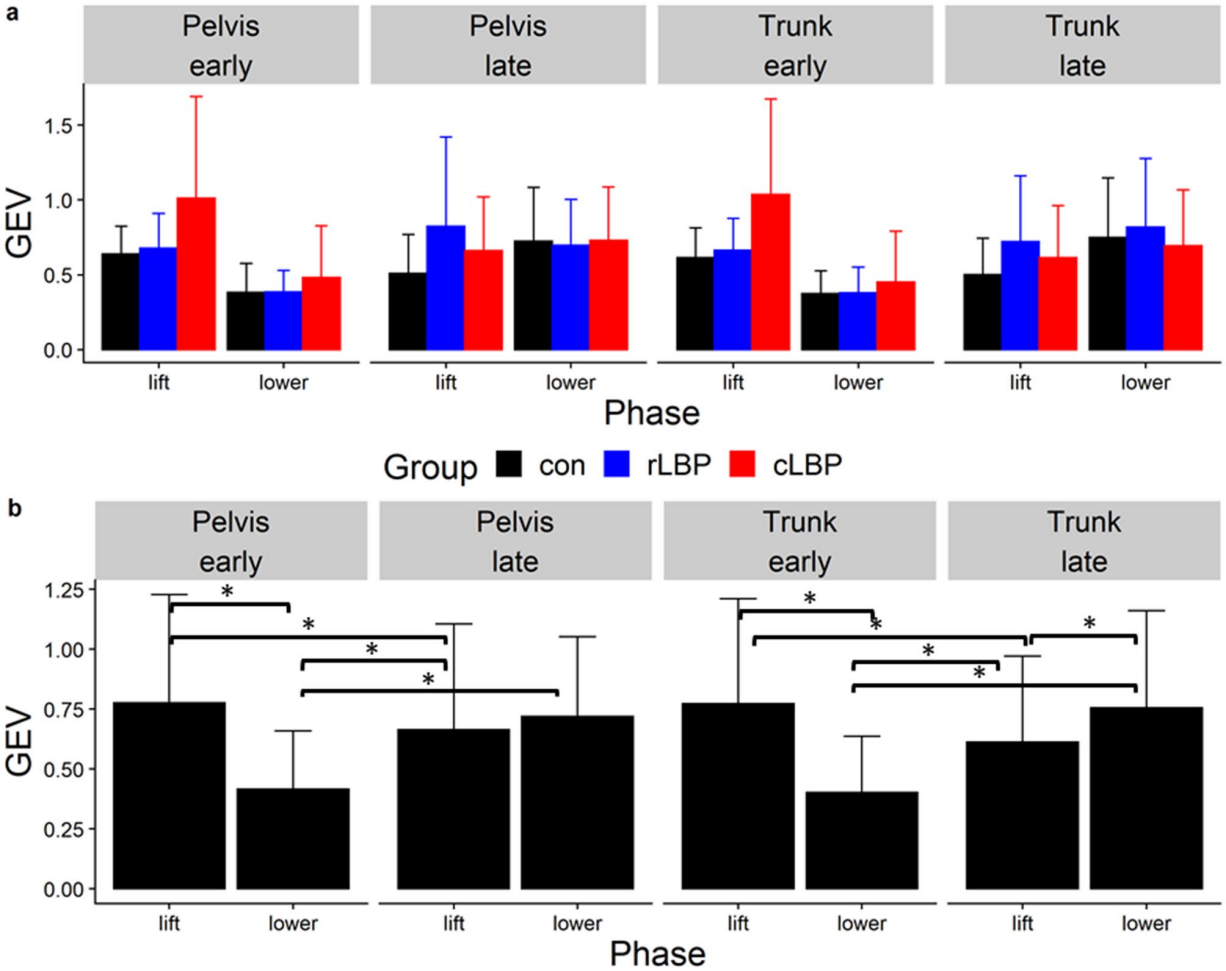
(**a**) Group mean (error represent one standard deviation [SD]) goal-equivalent variance normalized to the degrees of freedom (GEV) of each segment displacement, split by phase (lift, lower) and epoch (early, late); (**b**) mean (SD) GEV averaged across all participants split by segment displacement (pelvis, trunk) and phase (lift, lower) and epoch (early, late). *Con* control, *rLBP* remission low back pain, *cLBP* current low back pain. * indicates P < 0.05.

**Figure 6:**
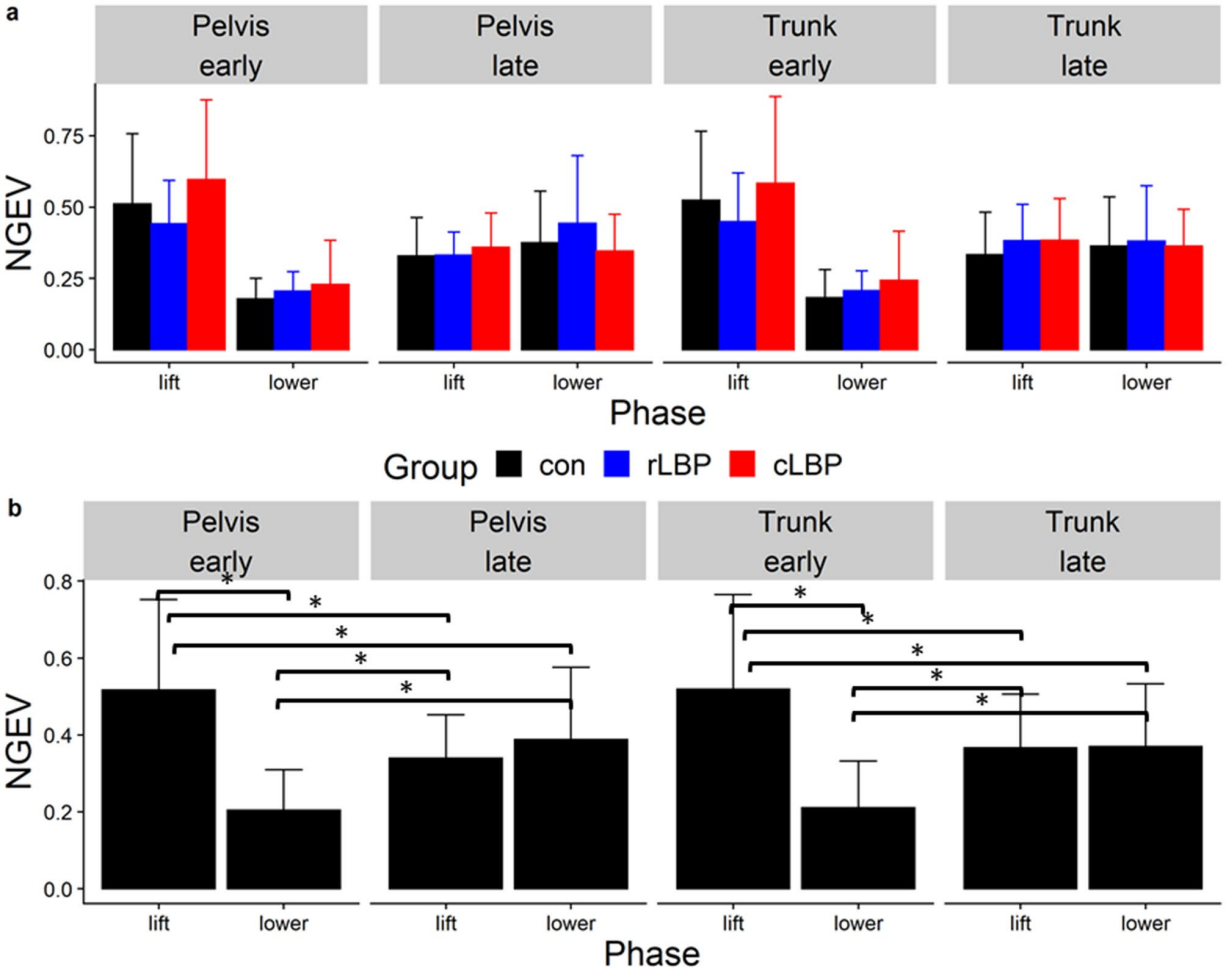
(**a**) Group mean (error as one standard deviation [SD]) non-goal equivalent variance normalized to the degrees of freedom (NGEV) of each segment displacement, split by phase (lift, lower) and epoch (early, late); (**b**) mean (SD) NGEV averaged across all participants split by segment displacement (pelvis, trunk) and phase (lift, lower) and epoch (early, late). *Con* control, *rLBP* remission low back pain, *cLBP* current low back pain. * indicates P < 0.05.

Across all participants, all three modes explained an average adjusted R^2^ value of between 0.85–0.89 of the pelvis and trunk displacements during lifting and lowering. The average root mean squared error (RMSE) of the observed and predicted displacements from the linear regressions to elicit the *J* matrices were between 0.05 to 0.16 m. The statistical values for all primary analyses can be found in the supplementary material. For all three dependent variables, there were no significant main effect of group, nor interactions between group and phase/epoch. For IMA, there was a significant main effect of phase for pelvis (${\chi }^{2} = 10.03$, P = 0.004) and trunk displacements (${\chi }^{2} = 15.03$, P = 0.001) (Fig. 4). There was a significant phase-by-epoch interaction for GEV (pelvis-${\chi }^{2} = 15.63$, P < 0.001; trunk-${\chi }^{2} = 14.47$, P = 0.001) (Fig. 5), and NGEV (pelvis-${\chi }^{2} = 34.42$, P < 0.001; trunk-${\chi }^{2} = 35.88$, P < 0.001) (Fig. 6). There was a significant main effect of phase for GEV (pelvis-${\chi }^{2} = 12.26,$ P = 0.002; trunk-${\chi }^{2} = 9.75$, P = 0.004) (Fig. 5), and NGEV (pelvis-${\chi }^{2} = 55.96$, P < 0.001; trunk-${\chi }^{2} = 60.73$, P < 0.001) (Fig. 6). There was a significant main effect of epoch for NGEV (pelvis-${\chi }^{2} = 9.26$, P = 0.017; trunk-${\chi }^{2} = 10.67$, P = 0.016) (Fig. 6). IMA was significantly greater during lowering than lifting for both pelvis (P = 0.001) and trunk displacements (P < 0.001) (Fig. 4). For both pelvis and trunk displacements, GEV and NGEV were greatest during the early lifting phase, and least during the early lowering phase (Figs. 5, 6).

## Discussion

The present study aimed to understand how muscles abundance may be altered during lifting between individuals with and without current LBP, and also those in LBP remission. Contrary to our first hypothesis, current or previous LBP had no influence on motor abundance, GEV, and NGEV. In contrast to our second hypothesis, muscle modes in cLBP reflecting a dominant ankle extensor and upper limb flexor pattern, rather than a dominant trunk extensor pattern, showed the largest increase in activity compared to controls (Fig. 3).

Findings from the present study are consistent with McCaskey et al.^19^, who also reported similar magnitudes of motor abundance, GEV, and NGEV between individuals with chronic LBP compared to asymptomatic controls. However, another study reported that individuals with chronic LBP had lower GEV, and higher NGEV, than asymptomatic controls^20^. Differences between studies could be attributed to several factors. First, the present study investigated used muscle modes, rather than joint angles as input DOFs into the UCM analysis^19,20^—meaning, how joint angles vary across task repetitions to stabilize a movement goal. Second, all three studies investigated motor control on different tasks. It is likely that motor abundance is heterogeneously affected in different tasks by the presence of LBP^44^. Third, LBP participants in the present study had greater pain intensity, compared to prior studies (VAS = 1.09/10^20^; VAS = 2.89/10^19^), and further investigations are required to determine the relationship between pain intensity, motor task difficulty, and motor abundance.

When a task is made more demanding, asymptomatic individuals appear to increase their GEV and NGEV^45,46^. The present findings were consistent with prior studies^45,46^, in that we found greatest GEV and NGEV during the early epoch of lifting. This is a period where the load is furthest from the spine, requiring the greatest joint torque to support the load. Since NGE deviations would perturb the pelvis and trunk positions, it is surprising that NGEV was not reduced during the early epoch of lifting. This finding can be interpreted in the context of previous studies showing a positive relationship between force demands in motor tasks and sensorimotor noise^47,48^. It is possible that the greater force demands during the early epoch of lifting increased sensorimotor noise arising from the generation of a motor command and muscular activation. Therefore, the increase in GEV may be a compensatory mechanism for the increase in NGEV, to preserve motor abundance in a period of lifting where force demands are high. The present findings that NGEV was similar between individuals with and without LBP does not imply that feedback and practice training have no role in the management of the disorder. The variability of spinal loads in asymptomatic individuals has been reported to be greater when lifting heavier (27 kg) than lighter (13 kg) loads^10^. Hence, the influence of LBP on muscle abundance may manifest during more challenging motor tasks, such as lifting a heavier load.

Although overall motor abundance was similar between groups, the activation profiles of muscle modes differed. Both cLBP and rLBP individuals had significantly greater activity of mode 2 (hip-knee extensor pattern) during the middle of the lifting phase compared to controls, which was consistent with prior studies which investigated hamstring activity^49,50^. In addition, both cLBP and rLBP individuals had significantly greater activity of mode 3 (lumbopelvic coactivation) during the late epoch of the lifting phase compared to controls, again consistent with previous studies investigating abdominal activity^6,51^. It would be expected that mode 3 activity would not be high during the late epoch of the lifting phase, given that the external lever arm of the load is lowest, thereby requiring little supportive torque. Given that mode 3 in lifting reflects lumbopelvic coactivation, spinal loads during lifting in individuals with cLBP and rLBP, may be adversely greater than in controls.

In the lowering phase, individuals with cLBP and rLBP had significantly greater activity of mode 1 (upper limb flexor pattern) during the early epoch, and greater activity of mode 3 (mixed multi-segmental pattern) during the late epoch, compared to controls (Fig. 2). Greater activity of mode 1 in lowering could have been a strategy to flex the elbow to keep the external load closer towards the body, thereby reducing the load’s lever arm, and consequently spinal tissue loading. Greater activity of mode 3 in the late epoch of lowering could have been a generalized co-activation, protective strategy in individuals with LBP and those in remission. Previous studies have reported that individuals with LBP either had an absent relaxation, or delayed relaxation, of the lumbar erector spinae muscles during trunk flexion, compared to controls^21,52^.

The present study has some limitations. First, a general limitation of the UCM analysis is that the variability of the residuals of the regression equations to attain the Jacobian, is not included when evaluating the variability within the GE and NGE subspaces. The extent to which the magnitude of residuals in the elicitation of the Jacobian impacts on the resultant UCM analysis, should be investigated in future research. Second, the present UCM analysis did not account for different and competing cost functions associated with different muscle mode activation combinations^53,54^. For example, at an instance in lifting, there could be a combination of muscle modes activation which results in the least spinal load. UCM analysis which accounts for different cost functions may further shed light on the complexities of motor control impairments with pain. Third, when investigating motor control variability in a clinical pain cohort, researchers will always have to balance the number of repetitions that can be performed to enable a stable estimate of variance measures, without excessive risk of exacerbating pain. A variant of the UCM approached, termed “Motor Equivalence”^55^,  may be considered when investigating cohorts with greater pain severity, that markedly reduces the capacity of many repeated movement performances. Lastly, given the cross-sectional nature of this study, we are unable to infer that differences in motor control are a risk factor for LBP onset or its recurrence.

In conclusion, individuals with LBP, including those in remission, had similar overall motor abundance, but with different activation profiles of muscle modes than controls during a low load lifting task. Future studies investigating how LBP influences motor abundance during lifting tasks of varying physical demands are warranted. Such knowledge may benefit the design of feedback and practice training that specifically addresses motor control impairments that is both individual and task specific.

## Supplementary information

Supplementary file1

## References

[1] Global, regional, and national incidence, prevalence, and years lived with disability for 328 diseases and injuries for 195 countries, 1990–2016: A systematic analysis for the Global Burden of Disease Study 2016. *The Lancet* **390**, 1211–1259, https://doi.org/10.1016/S0140-6736(17)32154-2 (2017). 10.1016/S0140-6736(17)32154-2 PMC5605509 28919117

[2] Henschke N (2008). Prognosis in patients with recent onset low back pain in Australian primary care: inception cohort study. BMJ.

[3] MacDonald D, Moseley GL, Hodges PW (2010). People with recurrent low back pain respond differently to trunk loading despite remission from symptoms. Spine (Phila Pa 1976).

[4] MacDonald D, Moseley GL, Hodges PW (2009). Why do some patients keep hurting their back? Evidence of ongoing back muscle dysfunction during remission from recurrent back pain. Pain.

[5] Falla D, Gizzi L, Tschapek M, Erlenwein J, Petzke F (2014). Reduced task-induced variations in the distribution of activity across back muscle regions in individuals with low back pain. Pain.

[6] Suehiro T, Ishida H, Kobara K, Osaka H, Watanabe S (2018). Altered trunk muscle recruitment patterns during lifting in individuals in remission from recurrent low back pain. J. Electromyogr. Kinesiol..

[7] Hubley-Kozey C, Moreside JM, Quirk DA (2014). Trunk neuromuscular pattern alterations during a controlled functional task in a low back injured group deemed ready to resume regular activities. Work.

[8] Marras WS, Ferguson SA, Burr D, Davis KG, Gupta P (2005). Functional impairment as a predictor of spine loading. Spine (Phila Pa 1976).

[9] Arjmand N, Plamondon A, Shirazi-Adl A, Lariviere C, Parnianpour M (2011). Predictive equations to estimate spinal loads in symmetric lifting tasks. J. Biomech..

[10] Granata KP, Marras WS, Davis KG (1999). Variation in spinal load and trunk dynamics during repeated lifting exertions. Clin. Biomech. (Bristol, Avon).

[11] Latash ML, Gorniak S, Zatsiorsky VM (2008). Hierarchies of synergies in human movements. Kinesiology (Zagreb, Croatia).

[12] Latash ML, Levin MF, Scholz JP, Schöner G (2010). Motor control theories and their applications. Medicina (Kaunas, Lithuania).

[13] Verrel J (2010). Distributional properties and variance-stabilizing transformations for measures of uncontrolled manifold effects. J. Neurosci. Methods.

[14] Srinivasan D, Mathiassen SE (2012). Motor variability in occupational health and performance. Clin. Biomech. (Bristol, Avon).

[15] Latash ML (2010). Motor synergies and the equilibrium-point hypothesis. Mot. Control.

[16] Marras WS, Davis KG, Ferguson SA, Lucas BR, Gupta P (2001). Spine loading characteristics of patients with low back pain compared with asymptomatic individuals. Spine (Phila Pa 1976).

[17] Ferguson SA, Marras WS, Burr DL, Davis KG, Gupta P (2004). Differences in motor recruitment and resulting kinematics between low back pain patients and asymptomatic participants during lifting exertions. Clin. Biomech. (Bristol, Avon).

[18] Lariviere C, Gagnon D, Loisel P (2002). A biomechanical comparison of lifting techniques between subjects with and without chronic low back pain during freestyle lifting and lowering tasks. Clin. Biomech. (Bristol, Avon).

[19] McCaskey MA, Wirth B, Schuster-Amft C, de Bruin ED (2018). Dynamic multi-segmental postural control in patients with chronic non-specific low back pain compared to pain-free controls: A cross-sectional study. PLoS ONE.

[20] Tajali S (2013). Multijoint coordination during sit-to-stand task in people with non-specific chronic low back pain. Biomed. Eng. Appl. Basis Commun..

[21] Murillo C (2019). High-density electromyography provides new insights into the flexion relaxation phenomenon in individuals with low back pain. Sci. Rep..

[22] von Elm E (2007). The strengthening the reporting of observational studies in epidemiology (STROBE) statement: Guidelines for reporting observational studies. PLoS Med.

[23] Stanton TR, Latimer J, Maher CG, Hancock M (2009). Definitions of recurrence of an episode of low back pain: A systematic review. Spine (Phila Pa 1976).

[24] Graham RB, Sadler EM, Stevenson JM (2012). Local dynamic stability of trunk movements during the repetitive lifting of loads. Hum. Mov. Sci..

[25] Schipplein OD, Reinsel TE, Andersson GB, Lavender SA (1995). The influence of initial horizontal weight placement on the loads at the lumbar spine while lifting. Spine (Phila Pa 1976).

[26] Zhou J, Dai B, Ning X (2013). The assessment of material handling strategies in dealing with sudden loading: Influences of foot placement on trunk biomechanics. Ergonomics.

[27] Asgari N, Sanjari MA, Esteki A (2017). Local dynamic stability of the spine and its coordinated lower joints during repetitive Lifting: Effects of fatigue and chronic low back pain. Hum. Mov. Sci..

[28] Hermens HJ, Freriks B, Disselhorst-Klug C, Rau G (2000). Development of recommendations for SEMG sensors and sensor placement procedures. J. Electromyogr. Kinesiol..

[29] Vera-Garcia FJ, Moreside JM, McGill SM (2011). Abdominal muscle activation changes if the purpose is to control pelvis motion or thorax motion. J. Electromyogr. Kinesiol..

[30] McGill S, Juker D, Kropf P (1996). Appropriately placed surface EMG electrodes reflect deep muscle activity (psoas, quadratus lumborum, abdominal wall) in the lumbar spine. J. Biomech..

[31] Vera-Garcia FJ, Moreside JM, McGill SM (2010). MVC techniques to normalize trunk muscle EMG in healthy women. J. Electromyogr. Kinesiol..

[32] Dempster W (1955). Space requirements of the seated operator: Geometrical, kinematic, and mechanical aspects of the body with special reference to the limbs.

[33] Hanavan E (1964). A mathematical model of the human body: Behavioural sciences laboratory.

[34] Drake JD, Callaghan JP (2006). Elimination of electrocardiogram contamination from electromyogram signals: An evaluation of currently used removal techniques. J. Electromyogr. Kinesiol..

[35] de Looze MP, Toussaint HM, van Dieen JH, Kemper HC (1993). Joint moments and muscle activity in the lower extremities and lower back in lifting and lowering tasks. J. Biomech..

[36] Kieliba P (2018). How are muscle synergies affected by electromyography pre-processing?. IEEE Trans. Neural Syst. Rehab. Eng..

[37] Gaujoux R, Seoighe C (2010). A flexible R package for nonnegative matrix factorization. BMC Bioinform..

[38] van den Hoorn W, Hodges PW, van Dieen JH, Hug F (2015). Effect of acute noxious stimulation to the leg or back on muscle synergies during walking. J. Neurophysiol..

[39] Ebied A, Kinney-Lang E, Spyrou L, Escudero J (2018). Evaluation of matrix factorisation approaches for muscle synergy extraction. Med. Eng. Phys..

[40] Liew, B., De Nunzio, A., Srivastava, S. & Falla, D. *Codes and Data to the paper "Influence of low back pain and its remission on motor abundance in a low-load lifting task"*, <https://github.com/bernard-liew/2020_lowbackpain_ucm_lifting> (2020). 10.1038/s41598-020-74707-4 PMC7576852 33082380

[41] Wood SN (2017). Generalized Additive Models An Introduction with R.

[42] Craven P, Wahba G (1978). Smoothing noisy data with spline functions. Numer. Math..

[43] Helwig NE, Shorter KA, Ma P, Hsiao-Wecksler ET (2016). Smoothing spline analysis of variance models: A new tool for the analysis of cyclic biomechanical data. J. Biomech..

[44] Dideriksen JL, Gizzi L, Petzke F, Falla D (2014). Deterministic accessory spinal movement in functional tasks characterizes individuals with low back pain. Clin. Neurophysiol..

[45] Scholz JP, Reisman D, Schöner G (2001). Effects of varying task constraints on solutions to joint coordination in a sit-to-stand task. Exp. Brain Res..

[46] Greve C, Hortobagyi T, Bongers RM (2015). Physical demand but not dexterity is associated with motor flexibility during rapid reaching in healthy young adults. PLoS ONE.

[47] Bays PM, Wolpert DM (2007). Computational principles of sensorimotor control that minimize uncertainty and variability. J. Physiol..

[48] Faisal AA, Selen LP, Wolpert DM (2008). Noise in the nervous system. Nat. Rev. Neurosci..

[49] Haddas R, Yang J, Lieberman I (2016). Effects of volitional spine stabilization on lifting task in recurrent low back pain population. Eur. Spine J.

[50] Kim MH, Yoo WG, Choi BR (2013). Differences between two subgroups of low back pain patients in lumbopelvic rotation and symmetry in the erector spinae and hamstring muscles during trunk flexion when standing. J. Electromyogr. Kinesiol..

[51] Yang HS (2018). Difference of the thickness and activation of trunk muscles during static stoop lift at different loads between subjects with and without low back pain. J. Back Musculoskelet. Rehab..

[52] Colloca CJ, Hinrichs RN (2005). The biomechanical and clinical significance of the lumbar erector spinae flexion-relaxation phenomenon: a review of literature. J. Manipulative Physiol. Ther..

[53] Togo S, Kagawa T, Uno Y (2016). Uncontrolled manifold reference feedback control of multi-joint robot arms. Front. Comput. Neurosci..

[54] Alessandro C (2020). Coordination amongst quadriceps muscles suggests neural regulation of internal joint stresses, not simplification of task performance. Proc. Natl. Acad. Sci..

[55] Furmanek MP, Solnik S, Piscitelli D, Rasouli O, Falaki A, Latash ML (2018). Synergies and Motor Equivalence in Voluntary Sway Tasks: The Effects of Visual and Mechanical Constraints. J. Mot. Behav..

