# Supplementary material for: Influence of low back pain and its remission on motor abundance in a low-load lifting task
Source: Sci Rep. 2020 Oct 20;10:17831. doi: 10.1038/s41598-020-74707-4 (PMC7576852; doi:10.1038/s41598-020-74707-4)
Supplement: Supplementary file 1 — Supplementary file1 [file 41598_2020_74707_MOESM1_ESM.pdf]

Supplementary material

Title: Influence of low back pain and its remission on motor abundance in a low-load lifting task.

Article type: Original Article

Bernard X.W. Liew, PhD [a]

Alessandro Marco De Nunzio, PhD[b]

Shraddha Srivastava[c]

Deborah Falla, PhD[d]

[a] School of Sport, Rehabilitation and Exercise Sciences, University of Essex, Colchester, Essex, CO4 3SQ, United Kingdom

[b] LUNEX International University of Health, Exercise and Sports, 50, avenue du Parc des Sports, L-4671 Differdange, Luxembourg

[c] Department of Health Sciences and Research, College of Health Professions, Medical University of South Carolina, 77 President Street, Charleston, SC, 29425, USA

[d] Centre of Precision Rehabilitation for Spinal Pain (CPR Spine), School of Sport, Exercise and Rehabilitation Sciences, University of Birmingham, Edgbaston B152TT, United Kingdom

Address correspondence and reprint requests to Dr Bernard Liew, School of Sport, Rehabilitation and Exercise Sciences, University of Essex, Colchester, Essex, CO4 3SQ, United Kingdom; E-mail: bl19622@essex.ac.uk, liew_xwb@hotmail.com

Source of funding: This study was supported by funding from the EUROSPINE Task Force Research pilot study grant (ID 2018_10) awarded to BXWL and DF.

Conflict of interest: All authors declare that they have no conflicts of interest.

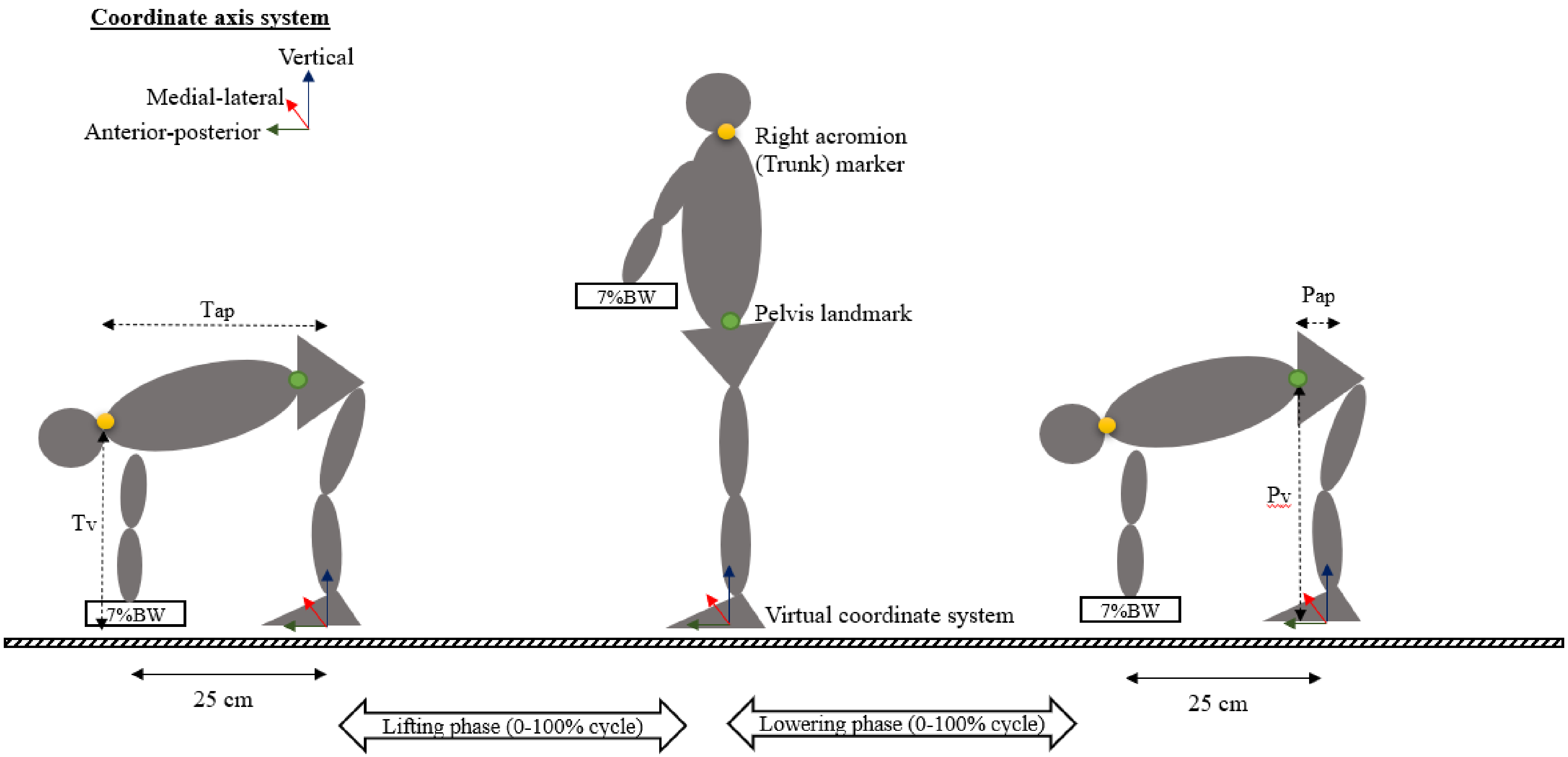

Figure 1. Schematic figure of the motor task. Abbreviations: Tv – trunk vertical displacement, Tap – trunk anterior-posterior displacement, Pv –pelvis vertical displacement, Pap – pelvis anterior-posterior displacement, BW – bodyweight

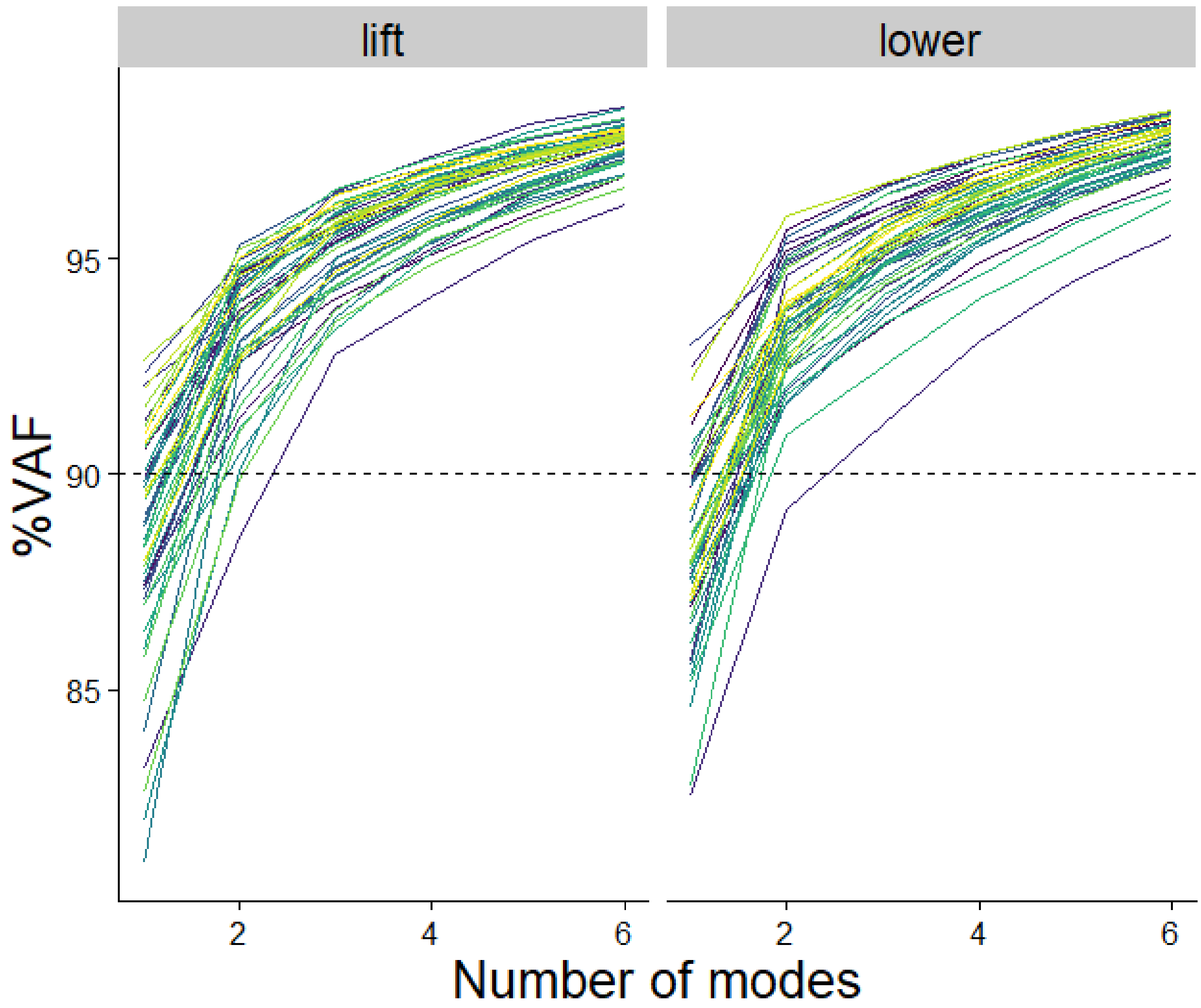

Figure 2 Percentage variance accounted for of the original electromyography signals relative to the number of modes extracted for each participant

Choice of statistical approach using GAM

The flexibility of GAM comes at a cost of losing the simplicity of reporting P-values in a traditional ANOVA analysis. Our reporting is identical to past studies using similar methods [1,2]. The question that comes down to why have a threshold of a non-zero 95%CI? The reason is that such a threshold is in the spirit of traditional P value testing – where a $P < 0.05$ threshold is actually equivalent to a non-zero 95%CI crossing, assuming the null hypothesis is that of no difference.

Traditional statistical methods are excellent if one has a prior hypothesis of where differences may lie. Pataky et al. termed it a “closed” hypothesis [3]. An example of a “closed” hypothesis is “there would be no group difference in peak spine flexion angle during lifting”. In our case, we left our hypothesis “open”, in that our hypothesis testing spans all % cycle of lifting and lowering. An example of an “open” hypothesis is “there would be no group differences in spine flexion angle during lifting”. The advantage of “open” hypothesis testing methods is that one can adopt a data-driven approach to understand where important differences lie. A disadvantage of using traditional statistics when one really have no strong “closed” hypothesis is one ends up inspecting the data, and cherry-picking datapoints for statistical inference, to maximize the chance of getting a favorable P-value – leading to false positives [4].

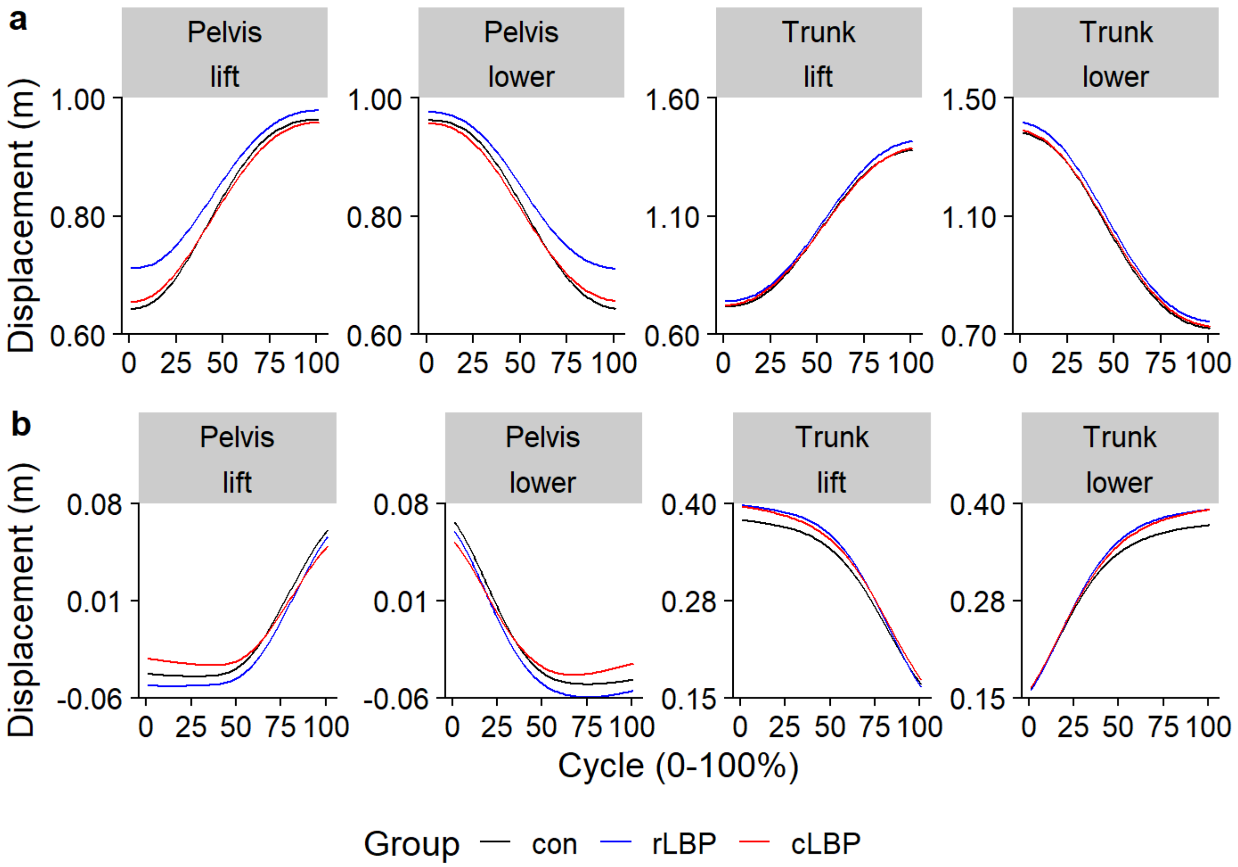

Figure 3 Group mean linear displacement (m) in the (a) vertical and (b) anterior-posterior direction (+ anterior to midpoint of foot) of the pelvis trunk wrist, during the lifting and lowering phases. Abbreviations: con – control, rLBP – remission low back pain, cLBP – current low back pain.

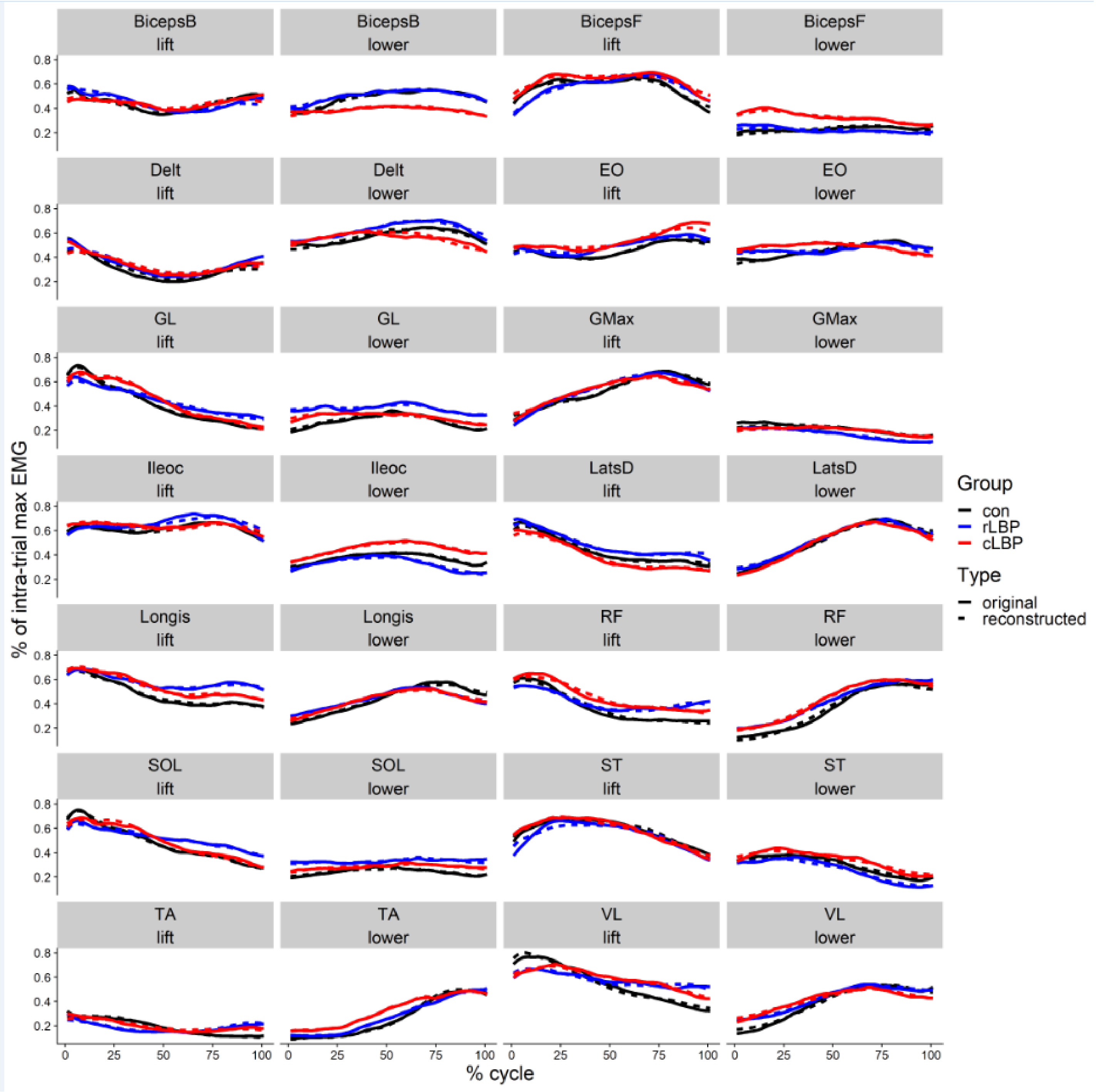

Figure 4 Group mean amplitude normalized (%) linear envelopes of the original and reconstructed EMG (from 3 modes) during lifting and lowering phases. Abbreviations: TA - tibialis anterior, SOL - soleus, GL - gastrocnemius lateralis, VL - vastus lateralis, RF - rectus femoris, BicepsF - biceps femoris, ST - semitendinosus, GMax - gluteus maximus, EO - external oblique, Longis - longissimus thoracis pars thoracis, Illeoc - iliocostalis lumborum, LatsD - latissimus dorsi, BicepsB - biceps brachii, Delt - deltoids, con – control, rLBP – remission low back pain, cLBP – current low back pain.

Table 1 Statistical results of primary mixed model analysis for IMA, GEV, and NGEV

No.	Kinematics	DV	χ^2	P	IV	P-adj
**1**	pelvic	IMA	0.825	0.662	group	1
**2**	pelvic	IMA	10.03	0.002	phase	0.004
**3**	pelvic	IMA	0.643	0.423	epoch	1
**4**	pelvic	IMA	1.07	0.586	group:phase	1
**5**	pelvic	IMA	1.461	0.482	group:epoch	1
**6**	pelvic	IMA	1.161	0.281	phase:epoch	0.689
**7**	pelvic	IMA	1.468	0.48	group:phase:epoch	1
**8**	trunk	IMA	3.105	0.212	group	0.848
**9**	trunk	IMA	15.028	0	phase	0.001
**10**	trunk	IMA	1.154	0.283	epoch	0.831
**11**	trunk	IMA	5.626	0.06	group:phase	0.882
**12**	trunk	IMA	1.731	0.421	group:epoch	1
**13**	trunk	IMA	2.033	0.154	phase:epoch	0.452
**14**	trunk	IMA	1.397	0.497	group:phase:epoch	1
**15**	pelvic	GEV	3.621	0.164	group	0.848
**16**	pelvic	GEV	12.255	0	phase	0.002
**17**	pelvic	GEV	3.115	0.078	epoch	0.38
**18**	pelvic	GEV	0.318	0.853	group:phase	1
**19**	pelvic	GEV	4.042	0.132	group:epoch	0.974
**20**	pelvic	GEV	15.635	0	phase:epoch	0
**21**	pelvic	GEV	1.762	0.414	group:phase:epoch	1
**22**	trunk	GEV	6.698	0.035	group	0.516
**23**	trunk	GEV	9.745	0.002	phase	0.004
**24**	trunk	GEV	2.523	0.112	epoch	0.412
**25**	trunk	GEV	2.001	0.368	group:phase	1
**26**	trunk	GEV	4.187	0.123	group:epoch	0.974
**27**	trunk	GEV	14.472	0	phase:epoch	0.001
**28**	trunk	GEV	0.5	0.779	group:phase:epoch	1
**29**	pelvic	NGEV	2.934	0.231	group	0.848
**30**	pelvic	NGEV	55.961	0	phase	0
**31**	pelvic	NGEV	9.26	0.002	epoch	0.017
**32**	pelvic	NGEV	2.014	0.365	group:phase	1
**33**	pelvic	NGEV	1.191	0.551	group:epoch	1
**34**	pelvic	NGEV	34.422	0	phase:epoch	0
**35**	pelvic	NGEV	0.73	0.694	group:phase:epoch	1
**36**	trunk	NGEV	1.841	0.398	group	1
**37**	trunk	NGEV	60.729	0	phase	0
**38**	trunk	NGEV	10.669	0.001	epoch	0.016
**39**	trunk	NGEV	2.582	0.275	group:phase	1
**40**	trunk	NGEV	2.475	0.29	group:epoch	1
**41**	trunk	NGEV	35.88	0	phase:epoch	0
**42**	trunk	NGEV	2.937	0.23	group:phase:epoch	1

Abbreviation: IMA – index of motor abundance, GEV – goal equivalent variance, NGEV – non-goal equivalent variance, DV – dependent variable, IV – independent variable.

References

1 Helwig, N. E., Shorter, K. A., Ma, P. & Hsiao-Wecksler, E. T. Smoothing spline analysis of variance models: A new tool for the analysis of cyclic biomechanical data. *J Biomech* **49**, 3216-3222, doi:https://doi.org/10.1016/j.jbiomech.2016.07.035 (2016).

2 Dixon, P. C., Stebbins, J., Theologis, T. & Zavatsky, A. B. Ground reaction forces and lower-limb joint kinetics of turning gait in typically developing children. *J Biomech* **47**, 3726-3733, doi:10.1016/j.jbiomech.2014.09.011 (2014).

3 Pataky, T. C., Vanrenterghem, J. & Robinson, M. A. Zero- vs. one-dimensional, parametric vs. non-parametric, and confidence interval vs. hypothesis testing procedures in one-dimensional biomechanical trajectory analysis. *J Biomech* **48**, 1277-1285, doi:10.1016/j.jbiomech.2015.02.051 (2015).

4 Pataky, T. C., Vanrenterghem, J. & Robinson, M. A. The probability of false positives in zero-dimensional analyses of one-dimensional kinematic, force and EMG trajectories. *J Biomech* **49**, 1468-1476, doi:10.1016/j.jbiomech.2016.03.032 (2016).
